# Advancing epilepsy genetics in the genomic era

**DOI:** 10.1186/s13073-015-0214-7

**Published:** 2015-08-25

**Authors:** Candace T. Myers, Heather C. Mefford

**Affiliations:** Department of Pediatrics, Division of Genetic Medicine, University of Washington, Seattle, WA 98195 USA

## Abstract

Epilepsy is a group of disorders characterized by recurrent seizures, and is one of the most common neurological conditions. The genetic basis of epilepsy is clear from epidemiological studies and from rare gene discoveries in large families. The three major classes of epilepsy disorders are genetic generalized, focal and encephalopathic epilepsies, with several specific disorders within each class. Advances in genomic technologies that facilitate genome-wide discovery of both common and rare variants have led to a rapid increase in our understanding of epilepsy genetics. Copy number variant and genome-wide association studies have contributed to our understanding of the complex genetic architecture of generalized epilepsy, while genetic insights into the focal epilepsies and epileptic encephalopathies have come primarily from exome sequencing. It is increasingly clear that epilepsy is genetically heterogeneous, and novel gene discoveries have moved the field beyond the known contribution of ion channels to implicate chromatin remodeling, transcriptional regulation and regulation of the mammalian target of rapamycin (mTOR) protein in the etiology of epilepsy. Such discoveries pave the way for new therapeutics, some of which are already being studied. In this review, we discuss the rapid pace of gene discovery in epilepsy, as facilitated by genomic technologies, and highlight several novel genes and potential therapies.

## The genetic basis of epilepsy

Epilepsy is defined by recurrent, unprovoked seizures due to abnormal, synchronized neuronal firing in the brain. The condition affects up to 1 in 26 individuals in the United States [1], making it one of the most common neurological conditions. Approximately 20–30 % of epilepsy cases are caused by acquired conditions such as stroke, tumor or head injury, but the remaining 70–80 % of cases are believed to be due to one or more genetic factors [2]. Although the definition of epilepsy suggests that it is a single disorder, it is more accurate to describe epilepsy as a group of disorders with diverse etiologies and outcomes. There are many different epilepsy syndromes that can be characterized by the seizure type(s), age of onset, developmental status, co-morbid features and etiology [3].

The epilepsies can be broadly grouped into three classes: genetic generalized epilepsy (GGE; formerly idiopathic generalized epilepsy); focal epilepsy; and epileptic encephalopathy (EE). There are then several specific syndromes within each class defined by differences in specific seizure types, electroencephalogram (EEG) patterns, age of onset and disease progression. We will refer to these as primary epilepsy disorders. The GGE syndromes, characterized by generalized seizures that involve both sides of the brain, include juvenile myoclonic epilepsy and childhood absence epilepsy among others. The GGEs tend to start in childhood or adolescence and are usually associated with normal development and intellect. Focal seizures originate in one hemisphere of the brain. Examples of focal epilepsy syndromes are temporal lobe epilepsy, autosomal dominant nocturnal frontal lobe epilepsy (ADNFLE), and autosomal dominant epilepsy with auditory features. The EEs are severe, early onset conditions characterized by refractory seizures, developmental delay or regression associated with ongoing epileptic activity, and generally poor prognosis. Dravet, Ohtahara and West syndromes are some of the most well-studied EEs. Importantly, epilepsy is often a co-morbid condition in individuals with intellectual disability (ID), autism or schizophrenia and may be a feature of many metabolic conditions and genetic syndromes. The focus of this review will be on the primary epilepsies, but we will also highlight some of the advances that increase our understanding of related neurodevelopmental conditions.

A genetic basis for epilepsy has been hypothesized for decades, but the first evidence of a genetic component emerged from epidemiological studies that reported an increased risk of epilepsy in relatives of affected individuals [4, 5]. Studies of twins showed that monozygotic twins have a higher concordance rate for both GGE and focal epilepsy than dizygotic twins, supporting the hypothesis that epilepsy has a genetic basis [6, 7]. Human genetics approaches that applied linkage analysis and gene mapping in large families in the 1990s yielded a steady trickle of gene discoveries, beginning with the finding that mutations in *CHRNA4*, encoding a subunit of the nicotinic acetylcholine receptor, cause ADNFLE [8]. Mutations in other genes encoding ion channels, such as *KCNQ2* in benign familial neonatal seizures [9] and *SCN1A* in Dravet syndrome [10], led to the “channelopathy” hypothesis, which postulates that dysfunction or dysregulation of ion channels is a common mechanism underlying epilepsy syndromes.

The emergence of genomic technologies, including chromosome microarrays and next-generation sequencing, has accelerated our understanding of the genetic architecture of the epilepsies, and the trickle of gene discoveries has increased to a flood. Importantly, these largely unbiased approaches have led to gene discoveries that expand our knowledge of critical pathways for epileptogenesis beyond the ion channels, opening the door for the development of novel therapies. These technologies have led to advances in understanding of the different genomic and genetic architectures across all the major classes of epilepsy, including uncovering a surprising overlap among seemingly different disorders. We will discuss the major advances in epilepsy genomics and genetics that have surfaced largely due to advances in these technologies.

## Copy number variants in epilepsy

Copy number variants (CNVs) are deletions and duplications of stretches of DNA ranging from 1 kilobase to an entire chromosome. CNVs are an important source of normal genomic variation, but some act as risk factors or causes of disease. The development of chromosome microarrays—which include comparative genomic hybridization and SNP genotyping arrays—allowed genome-wide screening for CNVs in large cohorts. CNV studies have been carried out for GGE, focal epilepsy and EE, revealing a clear role for CNVs in each major class. Overall, rare CNVs, some of which involve known disease genes, contribute to 5–10 % of cases of childhood epilepsies [11, 12].

Most studies of GGE suggest a complex genetic architecture with oligogenic inheritance [13, 14]. A few highly penetrant genetic risk variants have been identified, most through candidate gene studies. A major breakthrough in identifying genetic risk factors for GGE came with the application of genome-wide chromosome microarrays for genome-wide CNV detection in large cohorts of patients, the first of which revealed that large, recurrent deletions at chromosomes 15q13.3, 16p13.11 and 15q11.2 are significantly enriched in patients with GGE [15–18]. Each of these microdeletions is consistently found in 0.5–1 % of patients, with the 15q13 microdeletion conferring the most significant risk for epilepsy (odds ratio of 68) [19]. Consistent with the complex genetic architecture of the disorder, each deletion exhibits variable inheritance patterns (de novo or inherited) and incomplete penetrance [20]. The 15q13.3 deletion appears to be a risk factor primarily for GGE, while the 15q11.2 and 16p13.11 deletions are also found in patients with focal and other epilepsies [21]. Notably, all three deletions are known risk factors for ID, autism and schizophrenia as well, highlighting a shared genetic susceptibility for these disorders. Importantly, patients with GGE and ID are more likely to have one of these three deletions than patients with GGE and normal intellect [22]. Rare deletions involving the exons of some neuronal genes have recently been shown to increase risk for GGE, including *NRXN1* [23], *RBFOX1* [24] and *GPHN* [25].

To our knowledge, the largest study of patients with focal epilepsy carried out so far included 3812 affected individuals [21]. The 16p13.11 deletion described above was found in 23 cases (0.6 %). In addition, there was an excess of large (>2 megabase) rare deletions in cases compared to controls. In the EEs, pathogenic CNVs account for approximately 3–5 % of cases [11, 26]. Interestingly, deletions of 15q13.3, 15q11.2 and 16p13.11, important for GGE and focal epilepsy, are rarely seen in patients with EE, highlighting the notion that the major classes of epilepsy have different genetic architectures [27]. Importantly, identifying regions of overlap among non-recurrent deletions in similarly affected patients can point to novel disease genes. This approach prompted targeted sequencing of *CHD2*, a gene located in 15q26 deletions, and led to the discovery that de novo mutations cause an EE characterized by myoclonic seizures, photosensitivity and developmental delays [28, 29]. Targeted sequencing of *SLC6A1*, one of two genes in a minimally deleted region of 3p25 microdeletions, led to the discovery of de novo mutations in 4 % of patients with myoclonic astatic epilepsy [30]. *SLC6A1* encodes a GABA (γ-aminobutyric acid) transporter responsible for reuptake of GABA from the synapse. Conversely, the discovery of de novo mutations in *PURA* in patients with EE helped define *PURA* as the critical gene in patients with 5q31.3 deletions [31, 32].

## Genome-wide association studies

One of the earliest genomic approaches to complex disease was to perform genome-wide association studies (GWASs), designed to identify common genetic variants that contribute to risk of disease. Many GWASs have been carried out for epilepsy, although most of the early studies were too small to detect an effect or did not have replicable results. A recent effort by the International League Against Epilepsy produced a meta-analysis of 12 cohorts totaling >8000 cases with focal epilepsy or GGE and >26,000 controls [33]. The analysis identified polymorphic sequence variants as risk factors for the combined cohorts (focal and GGE) in *SCN1A*—perhaps the most well—known epilepsy gene—and in protocadherin 7 (*PCHD7*), a gene not yet known to be associated with epilepsy. In addition, when analyzing GGE alone, a signal at chromosome 2p16.1 emerged and was narrowed down to the interval containing the genes vaccinia-related kinase 2 (*VRK2*) and Fanconi anaemia, complementation group L (*FANCL*); there was no significant signal when evaluating focal epilepsies alone. The results from this study highlight the complex genetic architecture of focal epilepsy and GGE and the need for continued investigation in even larger cohorts.

Another recent GWAS comes from Feenstra and colleagues, in which they identify common genetic variants associated with risk for simple febrile seizures [34]. The study compared children who had a febrile seizure after receiving the measles–mumps–rubella (MMR) vaccine, children who had febrile seizures unrelated to the MMR vaccine, and controls without febrile seizures. Of four risk loci identified for febrile seizures overall, two were in well-known epilepsy genes (*SCN1A* and *SCN2A*). The variant associated with the highest risk of febrile seizures was in the anoctamin 3 (*ANO3*) gene, which encodes a transmembrane protein that belongs to a family of chloride channels, and the fourth locus was an intergenic region on chromosome 12q21.33. A particularly interesting aspect of this study was the identification of two variants, one in the interferon-induced gene *IFI44L* and the other in the measles virus receptor *CD46*, which are each specific for risk for febrile seizures after the MMR vaccine, suggesting that we may someday be able to identify a subset of children at risk for vaccine-related febrile seizures.

There have been no large GWASs performed for the EEs. Although these severe disorders are thought to be due largely to highly penetrant single-gene (or CNV) mutations, it is possible that GWASs of unsolved cases could reveal additional genetic risk factors.

## Massively parallel sequencing in epilepsy

The advent of next-generation (massively parallel) sequencing technology has revolutionized gene discovery in many disorders, including epilepsy. Applications of next-generation sequencing include gene panel, whole-exome and whole-genome sequencing. Gene panel and whole-exome sequencing are used most frequently for genetic testing in the clinic today and offer a rapid, affordable approach to mutation identification. Whole-genome sequencing is primarily used in the research laboratory, but will inevitably enter the clinical realm for diagnostics as it becomes more economical.

Perhaps the most significant recent advance in understanding the genetics of epilepsy has come from exome sequencing in the EEs, the most severe group of epilepsies. Because of the severity of disease, cases are usually sporadic with no other affected family members, precluding linkage analysis as a means to gene discovery. The discovery in 2001 that de novo mutations in *SCN1A* cause Dravet syndrome [10], one of the best-studied EEs, set the stage for the de novo paradigm for this class of disorders. Exome sequencing in single families and in both small and large cohorts has proven to be an essential tool to confirm the importance of de novo mutations, facilitate rapid gene discovery and highlight the genetic heterogeneity of this disorder. The major advances will be highlighted here, as a comprehensive discussion of the discoveries made is beyond the scope of this review.

In 2012, whole-genome sequencing in a single family with a severely affected child revealed a de novo *SCN8A* mutation in the proband [35]. Subsequent studies have confirmed the importance of this gene in the etiology of EE, with >25 cases reported with mutations in this gene since 2013 [36–39]. Most patients have a similar clinical presentation, with seizure onset at around five months of age and subsequent developmental delays and ID. In a slightly larger study, using exome sequencing in 39 patients with fever-associated epilepsies similar to Dravet syndrome, Nava and colleagues identified two de novo mutations in *HCN1* [40]. Sequencing *HCN1* in 157 additional affected individuals yielded four more patients with a mutation in this gene. HCN1 belongs to a family of hyperpolarization-activated, cyclic-nucleotide-gated channels that regulate neuronal excitability. Previous studies suggested that rare variants in *HCN1* and *HCN2* are risk factors for GGE [41]. Another study of 13 unsolved patients with Dravet syndrome revealed the importance of mutations in *GABRA1* and *STXBP1* [42], two genes previously implicated in other EEs. *GABRA1* encodes the α_1_ subunit of the GABA_A_ receptor, a multi-subunit chloride channel that serves as the receptor for the GABA inhibitory neurotransmitter. *STXBP1* encodes a syntaxin-binding protein that is critical for presynaptic vesicle docking and fusion. Mutations in *KCNB1*, a voltage-gated potassium channel, were also identified by exome sequencing in three families [43]. Other examples of recent gene discoveries are listed in Table 1.

**Table 1:** Novel epilepsy gene discoveries from 2012 to 2015

Gene	Phenotype(s)	Number of cases^a^	References
Chromatin remodeling			
*CHD2*	EOEE, LGS, EE, ASD	>20	[28, 44–46, 87]
Ion channels and neurotransmitter receptors			
*GABRA1*	DS, IS, JME, CAE, GGE	>10	[42, 46, 88–90]
*GABRB3*	IS, LGS	4	[46]
*GRIN2A*	LKS, CSWS, BECTS, ABPE, EE	>50	[91–97]
*GRIN2B*	IS, LGS, FE/ID, ID, ASD	>10	[46, 49, 54, 98]
*HCN1*	EOEE	4	[40]
*KCNB1*	IS	4	[43, 68]
*KCNA2*	EE	6	[99]
*KCNC1*	PME	13^b^	[100]
*KCNQ2*	BFNS, EOEE, EE	>50	[9, 72, 101–104]
*KCNT1*	MPSI, ADNFLE	14	[81, 105–108]
*SCN8A*	EE, EOEE	>30	[35, 37–39, 108]
*SLC6A1*	MAE	6	[30]
Intracellular signaling			
*GNAO1*	OS, IS, EE	6	[68, 109]
*SYNGAP1*	EE, ID, ASD	>20	[28, 54, 110, 111]
*TBC1D24*	MPSI, DOORS, EOEE, FE + ID, FIME, PME	>15	[100, 112–118]
Metabolism			
*CERS1*	PME	1^a^	[119]
*SLC13A5*	EOEE	3^a^	[120]
*SLC25A22*	NEESBs, MPSI, EME	4^a^	[121–124]
*SLC35A2*	EOEE, IS	8	[68, 125, 126]
Synaptic vesicle cycle			
*DNM1*	IS, LGS	5	[68]
*NECAP1*	EOEE	1^a^	[127]
*SNAP25*	EE	1	[128]
*STX1B*	Fever-associated epilepsy	6	[129]
*STXBP1*	EOEE, OS, IS, DS, EE	>50	[42, 130–137]
mTOR signaling			
*DEPDC5*	FFEVF, ADNFLE, BECTS, FCD, HME	>40	[58–64]
*MTOR*	FCD	18	[66, 138]
Multiple functions			
*ALG13*	IS, LGS	4	[46, 54, 134]
*EEF1A2*	IS, EOEE, ASD, ID, microcephaly	4	[54–56]
*PURA*	EOEE	15	[31, 32]
*WWOX*	EOEE, microcephaly	8^a^	[139–142]

*ABPE* atypical benign partial epilepsy, *ADNFLE* autosomal dominant nocturnal frontal lobe epilepsy, *ASD* autism spectrum disorder, *BECTS* benign epilepsy with centrotemporal spikes, *BFNS* benign familial neonatal seizures, *CAE* childhood absence epilepsy, *CSWS* continuous spike and waves during sleep, *DOORS* deafness, onychodystrophy, osteodystrophy, mental retardation and seizures syndrome, *DS* Dravet syndrome, *EE* epileptic encephalopathy, *EME* early myoclonic encephalopathy, *EOEE* early onset epileptic encephalopathy, FE focal epilepsy, *FFEVPF* familial focal epilepsy with variable foci, *FIME* familial infantile myoclonic epilepsy, *GGE* genetic generalized epilepsy, *HME* hemimegalencephaly, *ID* intellectual disability, *IS* infantile spasms, *JME* juvenile myoclonic epilepsy, *LGS* Landau–Kleffner syndrome, *MAE* myoclonic astatic epilepsy, *MPSI* migrating partial seizures of infancy, *NEESBs* neonatal epileptic encephalopathy with suppression bursts, *OS* Ohtahara syndrome, *PME* progressive myoclonus epilepsy.

^a^Refers to the number of families for recessive genes or isolated cases with respect to recurrent mutations.

^b^Unrelated probands have the same recurrent mutation (KCNC1 p.Arg320His), demonstrated to be de novo in 9/13.

Each of the discoveries described above adds to the growing list of genes implicated in epilepsy, and most support the channelopathy hypothesis. However, one of the distinct advantages of whole-genome technologies is the ability to use a hypothesis-free approach and discover the unexpected. In epilepsy, that includes genes involved in chromatin remodeling and transcriptional regulation, synaptic vesicle trafficking, and mammalian target of rapamycin (mTOR) signaling.

A growing class of genes implicated in epilepsy and related disorders are those that code for proteins involved in chromatin remodeling and transcriptional regulation. Through targeted sequencing of candidate genes from CNV regions, we identified 5 of 500 patients with EE who had a de novo mutation in *CHD2* [28]. This gene, which encodes chromodomain helicase DNA-binding protein 2, a chromatin remodeling factor, was also identified through exome sequencing in two Dravet-like cases [44]. To date, more than 20 patients with mutations in *CHD2* have been identified, with the majority of mutations being confirmed de novo events [28, 29, 44–47]. The epilepsy phenotype is characterized by multiple seizures types, primarily myoclonic, and exquisite photosensitivity in most patients [29]. In addition, variants in *CHD2* have been shown to be a risk factor for photosensitivity in the GGEs [48]. Targeted and whole-exome sequencing in a cohort of 580 patients with photosensitive epilepsy or photoparoxysmal response as determined by EEG revealed an overrepresentation of unique, either disruptive or predicted to be damaging, variants in *CHD2.* The classical photosensitive GGE—eyelid myoclonia with absences—had the highest frequency of unique variants, with *CHD2* explaining as many as 3 of 36 (8.3 %) cases. Notably, mutations have also been reported in patients with autism [49] and in ID without seizures [50]. Careful phenotyping of additional patients with mutations in *CHD2* will help define the phenotypic spectrum, which will undoubtedly be broader than that described for the first series of patients. CHD2 is ubiquitously expressed, and Encyclopedia of DNA Elements (ENCODE) data suggest that it acts as a transcriptional activator, although the target genes in the brain are not yet known. Mechanistic studies to identify the brain-specific function of CHD2 may yield novel drug targets for future therapies. Another gene that causes a surprisingly specific neural phenotype is the transcription factor myocyte enhancer factor 2C (*MEF2C*). While *MEF2C* has important regulatory roles in other tissue types such as cardiac and skeletal muscle, it is a critical gene in neural progenitor cell differentiation and maturation, and the causative gene in 5q14 deletion syndrome. Haploinsufficiency can cause a range of features, including hyperkinesis, variable epilepsy, ID and autism [51], as well as atypical Rett syndrome [52, 53].

*EEF1A2,* which encodes the α_2_ subunit of eukaryotic elongation factor 1, is another gene that merits further investigation. Recently, de novo mutations have been identified in four patients with severe early myoclonic epilepsy, hypotonia and developmental delay in three different studies [54–56]. The canonical role of EEF1A2 is translation elongation during protein synthesis, but diverse cellular functions, such as promotion of cell survival and growth, inhibition of apoptosis, and actin cytoskeletal remodeling via activation of the AKT pathway, have been reported [57].

Inroads have also been made in understanding the genetics of focal epilepsies through exome sequencing, revealing novel pathways beyond the ion channel. Two groups simultaneously reported mutations in *DEPDC5* in patients with familial focal epilepsy with variable foci (FFEVF) and other focal epilepsies [58, 59]. This gene has since been implicated in various childhood focal epilepsies [60] and ADNFLE [61] as well. Surprisingly, mutations have been described in patients with focal epilepsy and brain malformations, including bottom of the sulcus dysplasia, heterotopia, focal cortical dysplasia and hemimegalencephaly [62–64]. This is a particularly important finding, as epilepsy due to brain malformations has often been classified as “acquired”, or non-genetic rather than genetic, but now we must rethink traditional classification schemes. DEPDC5 is a member of the GATOR complex, a negative regulator of the mTOR pathway. mTOR is a serine/threonine kinase that converges multiple intracellular and extracellular signals to regulate cell growth, proliferation, survival, motility and metabolism. Dysregulation of mTOR signaling can cause a variety of diseases, including tuberous sclerosis (mutations in *TSC1*, *TSC2*), hemimegalencephaly (*AKT3*, *PIK3CA*, *MTOR*) and focal cortical dysplasia (*DEPDC5*, *AKT3*, *MTOR*) [65, 66]. Consistent neurological features of these disorders include intractable epilepsy, ID and autistic features, thus implicating these genes in epileptogenesis.

Exome-sequencing studies in GGE have been less successful at identifying clear genetic risk factors or high-penetrance mutations. In the largest study of GGE to date, exome sequencing in 118 affected individuals and 242 controls revealed several candidate sequence variants that were found only in affected cases [21]. However, follow-up genotyping of 3897 candidate variants in additional cases and controls failed to identify statistically significant differences between cases and controls. Very large cohorts may be required to make progress in GGE due to the complex genetic architecture that likely underlies this class of epilepsy.

## Genetic heterogeneity

With a few exceptions, most of the novel epilepsy genes that have been described recently are mutated in only a handful of patients (Table 1). While the numbers of affected individuals with mutations in a given gene will undoubtedly grow as additional patients are evaluated, it is clear that all classes of epilepsy are genetically heterogeneous. For the EEs, a recent, large trio exome study highlights this point [46].

The Epi4K consortium used trio exome sequencing to identify de novo mutations in patients with infantile spasms or Lennox–Gastaut syndrome (LGS) [46, 67], two classical EEs. In a study of 264 parent–child trios, a total of 329 de novo mutations in 305 genes were identified [46]. The majority of clearly causative mutations were in genes already known to cause EE, including *SCN1A*, *STXBP1*, and *CDKL5*, but two new disease genes emerged: *GABRB3* and *ALG13. GABRB3* encodes the β_3_ subunit of the GABA_A_ receptor, supporting the channelopathy hypothesis, while *ALG13* encodes a subunit of the uridine diphosphate *N*-acetylglucosamine (UDP-GlcNAc) transferase that is involved in N-linked glycosylation, an essential modification for protein folding and stability. Many candidate genes from this study will need to be sequenced across larger cohorts to identify additional de novo mutations. This is exemplified by the identification of *DNM1* as a causative gene of EE. The Epi4k analysis of 264 exomes identified two patients with de novo mutations in *DNM1* [46], but this number did not reach statistical significance given the size of the gene and predicted sequence-specific mutation rate. By analyzing another 92 cases in collaboration with the EuroEPINOMICS consortium, three additional de novo mutations were identified, solidifying the pathogenic role of mutations in *DNM1* [68]. *DNM1* encodes dynamin 1, a neural-specific GTPase that localizes to the presynaptic terminal and is important for the scission of synaptic vesicles from the plasma membrane.

Mutations in numerous genes can also cause focal epilepsy syndromes, including the recently described *DEPDC5*, *KCNT1* and *GRIN2A* genes, and a number of genes have been identified as risk factors for or causes of GGE. However, for both classes of epilepsy, the genes identified to date only explain a fraction of cases, suggesting that there is also considerable genetic heterogeneity in focal epilepsy and GGE.

Recent gene discoveries such as *GABRB3*, *GRIN2A*, *HCN1*, *KCNA2*, and *KCNH1* support the channelopathy hypothesis, and genes that encode ion channels certainly represent the largest class of disease-causing genes in epilepsy. However, other recent gene discoveries, including *ALG13*, *EEF1A2*, *GNAO1*, *NECAP1*, *SNAP25*, *STX1B*, *STXBP1*, *PURA*, and *WWOX*, highlight that epileptogenesis is caused by perturbations in diverse cellular pathways (Table 1), providing further evidence that epilepsy is not explained wholly as a channelopathy.

## Expanding the phenotypic spectrum

There is an increasingly wide phenotypic spectrum associated with mutations in *GABRA1*, *KCNQ2*, *KCNT1*, *SCN8A*, *TBC1D24*, and *DEPDC5*. Genetic modifiers and/or environmental risk factors may influence the epilepsy subtype or severity; this will require more systematic studies in the future. Careful experiments evaluating the functional consequences of specific dominant mutations are beginning to explain some of these differences [69–71]. A recent example comes from the potassium channel subunit Kv7.2, encoded by KCNQ2, in which alterations at the same amino acid (p.Arg213Trp and p.Arg213Gln) lead to strikingly different phenotypes [69]. The Arg213Trp change caused benign familial neonatal seizures, a dominantly inherited neonatal epilepsy with a generally favorable prognosis, in contrast to the Arg213Gln change, which caused neonatal EE with severe pharmacoresistant seizures, EEG consistent with a burst-suppression pattern, macrocephaly, and neurocognitive and motor delays [69, 72]. Miceli and colleagues demonstrated that both mutations in the potassium channel subunit Kv7.2 caused a decrease in channel voltage sensitivity, but the Arg213Gln mutation had a more dramatic effect, likely explaining the more severe phenotype. Detailed mutation modeling such as this will be required to understand how molecular perturbations underlie the etiology of epilepsy syndromes.

For some genes, the phenotypic spectrum expands beyond the epilepsies to other neurodevelopmental disorders, including autism and ID. For example, although the majority of patients with mutations in *STXBP1*, SYNGAP1, or *CHD2* present with seizures, mutations have also been identified in individuals with ID and/or autism spectrum disorder but without epilepsy [49]. Mutations in *SCN1A* have been found in patients from autism cohorts [73], and mutations in *SCN2A* cause a range of neurodevelopmental conditions with and without seizures [74]. It is important to remember that there may be selection and phenotyping bias in large cohorts; that is, phenotype information for patients recruited to “autism” studies will focus on autistic features and subtypes whereas patients in “epilepsy” cohorts will likely have detailed seizure and EEG information available. Careful and comprehensive phenotyping of patients *after* a mutation is identified will help determine the full phenotypic spectrum associated with a given mutation within a gene, without such phenotypic bias.

## Where are the missing mutations?

Genomic technologies have afforded tremendous progress in understanding the genetics of epilepsy, but there is still much work to be done. Despite whole-exome sequencing in trios, a large number of cases of EE remain unsolved. The combined Epi4K/EuroEPINOMICS study conservatively estimates that trio sequencing provided a clear genetic etiology in up to ~12 % of 356 samples. Most cases had at least one de novo coding variant; for a subset of cases, the de novo variant may be causative, although this is difficult to prove without additional cases or functional studies. Recessive mutations may explain a minority of cases but are more likely in families where consanguinity is present. Post-zygotic, somatic mosaic mutations are increasingly recognized as an important cause of disease across a range of disorders. In the epilepsies, many of the mutations in the mTOR pathway that have been found in patients with brain malformations are somatic mosaic mutations [75–77]. Individuals with somatic mutations may present with variable phenotypes and severity depending on how many and which cells carry the mutation. Importantly, somatic mosaic mutations are not always detectable in DNA from blood, although increasingly low levels of mosaicism can be detected through deep next-generation sequencing [76]. In some cases, the mutation may be present only in the affected tissue, which will be difficult to obtain for many epilepsies. Resection of affected tissue in focal epilepsies may shed light on how this mechanism may help define the role of mosaic mutations in epilpesy. Finally, a largely unexplored area is the impact of mutations in non-exonic DNA. Rare noncoding mutations have been identified in some human diseases and are likely to play a role in epilepsy as well. Whole-genome sequencing in trios will reveal candidate de novo variants for further exploration of this hypothesis. Sorting out the complex genetics of GGE will likely require much larger cohorts and families to understand the combination of factors that contribute to genetic risk.

## Clinical implications

Establishing the genetic basis of epilepsy in a given patient is important for counseling with respect to disease prognosis, for determining recurrence risk for future pregnancies and for treatment decisions in select cases [78]. A genetic diagnosis ends the diagnostic odyssey and eliminates unnecessary medical tests, and can provide the family with the opportunity to identify others in a similar situation and connect with support groups. There has been a recent growth in family organizations for the EEs, such as the Dravet Foundation, LGS Foundation, PCDH19 Alliance, Jack Pribaz Foundation (for those with *KCNQ2* mutations) and FAMILIEScn2A (*SCN2A* mutations), among others. These organizations are powerful patient advocates, providing information and support to affected families; in addition, some family organizations play an important role in funding research.

The genetic heterogeneity of epilepsy influences genetic testing, as testing one gene at a time is no longer a practical approach. The development of gene panels and the introduction of exome sequencing for clinical diagnostics now provide more comprehensive and affordable options for testing and should be implemented early in the diagnostic process. Chromosome microarrays should be considered in severe cases and in GGE with co-morbid features, where the likelihood of finding a disease-associated CNV is highest.

Beyond diagnostics, a major goal of genetic studies is to identify novel drug targets and to be able to make treatment choices based on the genetic cause of disease—an approach termed precision medicine. This will not be a simple task given the genetic complexity of epilepsy, in which mutations in different genes cause syndromes that are clinically indistinguishable, and mutations in a single gene, such as *SCN1A*, can cause a range of phenotypes varying from febrile seizures to severe EEs. However, recent reports offer the first insights into the power of precision medicine in treating epilepsy [79, 80]. Exome sequencing identified a heterozygous missense mutation in *KCNT1* (c.1283G > A; p.Arg428Gln) in a child with migrating partial seizures of infancy (MPSI); the same mutation had been previously reported in three other patients with MPSI. Functional studies in *Xenopus* oocytes demonstrated that the mutant protein caused a hyperactive channel [81], and that the effects of the mutation could be partially reversed by quinidine, an antiarrhythmic drug that is a partial antagonist of the potassium channel encoded by KCNT1 [70, 82]. Administration of oral quinidine in this child correlated with diminished or absent seizure activity and an improvement in psychomotor skills. Similarly, the administration of memantine, a US Food and Drug Administration-approved NMDA (*N*-methyl-d-aspartate) receptor antagonist, greatly reduced seizure activity in a patient with early onset EE caused by a de novo missense mutation in *GRIN2A* (p.Leu812Met) [80]. In vitro experiments testing for activity of the mutant receptor were performed prior to administration of the drug, an important point as not all mutations affect a protein equally. In vitro experiments performed on another GRIN2A mutant protein (p.Asn615Lys), which was the genetic cause of another child’s early onset EE, demonstrated that this mutation had a different effect on protein function entirely. *GRIN2A* encodes a subunit of the NMDA receptor, which is an ion channel that is activated by glutamate. Under normal conditions of synaptic transmission, very few ions pass through the channel because Mg^2+^ blocks the pore. Upon receptor activation, Mg^2+^ is displaced, which allows Ca^2+^ and other cations to move into the cell. A dysfunctional NMDA receptor can cause excess calcium influx into the cell and neurotoxicity. The Leu812Met change caused a hyperactive receptor, while the Asn615Lys mutation had no effect on receptor activity and acted through loss of the Mg^2+^ block. Although both mutations resulted in an increased flow of current, leading to excessive excitatory drive and early onset EE, it was important to consider the different pathological mechanisms and drug targets when deciding treatment options.

These studies highlight the importance of obtaining a molecular diagnosis early in development when possible, the use of cell culture and animal models to aid in the interpretation of the functional consequences of a mutation, and the need for rigorous clinical trials to standardize dosing and evaluate potential side effects of treatment. Other monogenic epilepsies for which a molecular diagnosis may influence the choice of anti-epileptic drugs are *KCNQ2*-related epilepsies and the use of ezogabine [83, 84], *SCN1A*-related Dravet syndrome and the use of clemizole [85], and *DEPDC5* and the use of the rapamycin derivative everolimus, which has already been effective in early clinical trials [86].

## Conclusions and future directions

Advances in DNA sequencing and interpretation of genetic variation are rapidly changing our understanding of the causes of epilepsy and its clinical management. It is unlikely that an individual gene will explain a large proportion of the GGEs, so efforts should be made to sequence large cohorts to gain broad insight into the mechanisms of epileptogenesis. Exome sequencing of trios has revealed the prominence of de novo mutations as a genetic cause of severe epilepsies, indicating that even in the absence of a family history, a genetic cause should be considered. Another important point to be taken away from the next-generation sequencing experiments is the diverse classes of genes that are emerging as being implicated in epilepsy. The recent surge of novel findings moves beyond the channelopathy hypothesis, implicating pathways that regulate synaptic vesicle trafficking, mTOR signaling, chromatin remodeling and transcriptional regulation, which offer new insights into disease-causing mechanisms and provide novel avenues for therapeutics. Despite recent advances, there are many genetic mysteries that remain to be unraveled. As whole-genome sequencing becomes more prevalent, the pace of discovery is likely to accelerate once again. Collaborations that include large cohorts and the sharing of research data will expedite gene discovery across all types of epilepsy, and central repositories for large-scale sequencing projects and epilepsy-specific findings will aid in the interpretation of rare variations. Finally, centralized variant databases and streamlined approaches to functional studies will move the field closer to translating genetic discoveries to directed therapies as we enter the era of precision medicine.

## Abbreviations

ABPE: Atypical benign partial epilepsy
ADNFLE: Autosomal dominant nocturnal frontal lobe epilepsy
ASD: Autism spectrum disorder
BECTS: Benign epilepsy with centrotemporal spikes
BFNS: Benign familial neonatal seizures
CAE: Childhood absence epilepsy
CNV: Copy number variation
CSWS: Continuous spike and waves during sleep
DOORS: Deafness, onychodystrophy, osteodystrophy, mental retardation and seizures syndrome
DS: Dravet syndrome
EE: Epileptic encephalopathy
EEG: Electroencephalogram
EME: Early myoclonic encephalopathy
ENCODE: Encyclopedia of DNA Elements
EOEE: Early onset epileptic encephalopathy
FE: Focal epilepsy
FFEVPF: Familial focal epilepsy with variable foci
FIME: Familial infantile myoclonic epilepsy
GGE: Genetic generalized epilepsy
GWAS: Genome-wide association study
HME: Hemimegalencephaly
ID: Intellectual disability
IS: Infantile spasms
JME: Juvenile myoclonic epilepsy
LGS: Landau–Kleffner syndrome
MAE: Myoclonic astatic epilepsy
MMR: Measles–mumps–rubella vaccine
MPSI: Migrating partial seizures of infancy
NEESBs: Neonatal epileptic encephalopathy with suppression bursts
OS: Ohtahara syndrome
PME: Progressive myoclonus epilepsy
